# Translocation pathways for inhaled asbestos fibers

**DOI:** 10.1186/1476-069X-7-4

**Published:** 2008-01-24

**Authors:** G Miserocchi, G Sancini, F Mantegazza, Gerolamo Chiappino

**Affiliations:** 1 Department of Experimental Medicine, University of Milano-Bicocca, Via Cadore 48, 20052, Monza, Italy; 2 Clinic of Occupational Medicine and Research Centre of Inhaled Particles, University of Milano, Via San Barnaba, 8 – 20122 Milano, Italy

## Abstract

We discuss the translocation of inhaled asbestos fibers based on pulmonary and pleuro-pulmonary interstitial fluid dynamics. Fibers can pass the alveolar barrier and reach the lung interstitium via the paracellular route down a mass water flow due to combined osmotic (active Na^+ ^absorption) and hydraulic (interstitial pressure is subatmospheric) pressure gradient. Fibers can be dragged from the lung interstitium by pulmonary lymph flow (primary translocation) wherefrom they can reach the blood stream and subsequently distribute to the whole body (secondary translocation). Primary translocation across the visceral pleura and towards pulmonary capillaries may also occur if the asbestos-induced lung inflammation increases pulmonary interstitial pressure so as to reverse the trans-mesothelial and trans-endothelial pressure gradients. Secondary translocation to the pleural space may occur via the physiological route of pleural fluid formation across the parietal pleura; fibers accumulation in parietal pleura stomata (black spots) reflects the role of parietal lymphatics in draining pleural fluid. Asbestos fibers are found in all organs of subjects either occupationally exposed or not exposed to asbestos. Fibers concentration correlates with specific conditions of interstitial fluid dynamics, in line with the notion that in all organs microvascular filtration occurs from capillaries to the extravascular spaces. Concentration is high in the kidney (reflecting high perfusion pressure and flow) and in the liver (reflecting high microvascular permeability) while it is relatively low in the brain (due to low permeability of blood-brain barrier). Ultrafine fibers (length < 5 μm, diameter < 0.25 μm) can travel larger distances due to low steric hindrance (in mesothelioma about 90% of fibers are ultrafine). Fibers translocation is a slow process developing over decades of life: it is aided by high biopersistence, by inflammation-induced increase in permeability, by low steric hindrance and by fibers motion pattern at low Reynolds numbers; it is hindered by fibrosis that increases interstitial flow resistances.

## Review

### Introduction

Asbestos fibers are known to be durable and not easily digested or dissolved after being inhaled into the lung. It was reported that asbestos fibers translocate from the lung into other tissue including pleural and peritoneal tissues [1,2]. Asbestos fibers translocated into the mesothelial tissue play an important role for the induction of asbestos related serosal disease, such as pleural and peritoneal fibrosis, as well as malignant pleural and/or peritoneal mesothelioma [3].

The aim of this paper is that of discussing the translocation of inhaled asbestos fibers from the lung to other body compartments based on knowledge of pulmonary and pleuro-pulmonary interstitial fluid dynamics [4-6]. Although it appears difficult to monitor the process of asbestos translocation as it takes place over decades of life, it appears useful to discuss of asbestos fibers as being dragged by bulk flows of water among compartments. We address this issue by considering the pressure gradients governing the inter-compartmental fluid exchanges, the physical features of the corresponding flows and the particular motion pattern of anisodiametric particles dragged by such flows in the tissues and across membranes delimiting the compartments.

Atmospheric asbestos pollution includes fibers whose length and diameter vary greatly according to dust formation process, distance from the source and the mineral variety of asbestos.

### The cellular impact of asbestos fibers

When inhaled mineral particles establish contact with biological tissues, reactions occur depending upon chemical as well as physical properties of the fibers. In the early pathogenic response, adsorption phenomena prevail in the cell-particle interactions [7]. Oxidation, as well as surface hydration and hydroxylation may occur in a moist atmosphere along the airways [8]. Studies on cultured A549 cells indicate that exposure to asbestos fibers initiate free radical reactions, inhibit glucose-6-phosphate dehydrogenase activity, decrease reduced glutathione and increase leakage of the cytoplasmatic enzyme lactate dehydrogenase, a sign of plasma membrane damage. These effects indicate an increase in cellular distress upon exposure to asbestos fibers [9]. Positively charged chrysotile fibers may bond to cell surfaces through a charge mediated effect, while amphibole fibers can bind to fibronectin [10]. Asbestos exposure can also stimulate gene expression via intracellular signaling (MAPK cascade responds to cell surface stimuli) that governs proliferation, apoptosis and inflammation [11]. Asbestos fibers may undergo phagocytosis by alveolar macrophages where the high concentration of oxidants and free radical release may induce chemical modifications at their surface and induce release of chemical mediators. Phagocytosis of crocidolite asbestos fibers by mesothelial cells was shown to induce intracellular oxidation, DNA strand breakage and apoptosis [12]. Inhaled asbestos fibers produce sclerogenic and carcinogenic effects on the lung parenchyma (asbestosis and carcinoma) [11].

Grinding and milling affect both the form and surface composition of fibers and were shown to cause an increase in reactive oxygen species [13]. Amphibole asbestos are the most biopersistent particles *in-vivo*, a property reflecting their low solubility [14]. Solubility is increased by leaching, a process of progressive splitting of chrysotile bundles of fibers into fibrils occurring in cells, particularly in alveolar macrophages. Leaching causes an increase in chemically active surface area, and facilitates penetration of solvent molecules [14].

### Fibers transport across the alveolar surface

**Figure 1:**
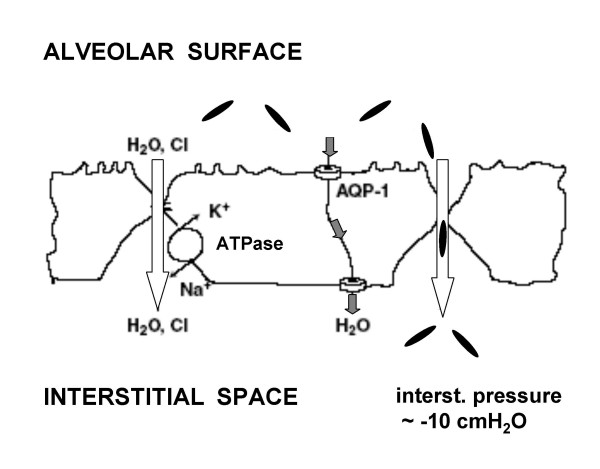
Uptake of asbestos fibers (in black) via the paracellular pathway down the physiological water absorption is favoured by the subatmospheric interstitial pressure and by the osmotic gradient generated in the baso-lateral spaces by the activity of Na^+^/K^+^ ATP-ase pump. A death of alveolar epithelial cells after asbestos fibers phagocytosis followed by desquamation of the alveolar lining may represent another translocation pathway. Transcellular water through aquaporins (AQP-1) is also shown.

Inhaled anisodiametric fibers, even of larger dimensions (length up to tens of μm, diameter up to 1–2 μm), remain oriented parallel to the airflow direction in the upper respiratory tract and can reach the alveoli along with progressively smaller fibers down to ultrefine and ultrashort fibers (length < 5 μm, diameter < 0.25 μm). Both microscopic and ultramicroscopic size fibers are found in the alveoli of subjects occupationally exposed to asbestos. Ultrafine fibers (diameter of 0.02–0.2 μm, and length of a few μm), can travel large distances from their source and can be recovered in subjects exposed to general environmental pollution [15]. A potential route for uptake of asbestos fibers is via the paracellular pathway down the gradient for physiological water absorption, as schematically shown in Fig. 1. This flow is aided by the existence of a subatmospheric hydraulic pressure in the interstitial space[6] and by the osmotic gradient generated in the baso-lateral spaces due to the activity of Na^+^/K^+^ ATP-ase. The fibers cause direct cellular damage by affecting the epithelial cellular layer (death of alveolar cells and desquamation of the alveolar lining). Fenestrations are other possible routes through which fibers are able to reach the lung interstitium. Alveolar macrophages can contribute, after asbestos fibers phagocytosis, to their subsequent translocation to either the extracellular space or the blood. Macrophages were shown to phagocytose asbestos fibers of considerable length and one can hypothesize that fibers' translocation across compartments is aided by the macrophages ability to move across barriers. Asbestos fibers accumulation in macrophages could still be demonstrated two years after a 6 weeks exposure to amosite [16].

An experimental model in rats has compared the pathways for translocation of insoluble particles instilled intratracheally [17]: crocidolite asbestos fibers were found to move relatively easily through the epithelial layer as their lung interstitial burden increased sharply 24 h after inhalation, compared to inert polystyrene microspheres of the same size. Conversely, phagocytosis by alveolar macrophages remained quite low for crocidolite while it increased markedly for inert polystyrene microspheres. One would deduce from these findings that the contribution of macrophages to crocidolite fibers translocation from alveoli to lung interstitium is marginal. Phagocytosis was also demonstrated for epithelial cells. The persistence of asbestos fibers in the alveolar compartment is increased in chronic lung inflammation because of reduced clearance mechanisms through the airways [18].

### Motion of particulate in fluid at low Reynolds number

Translocation of inhaled asbestos fibers to extrapulmonary sites has been demonstrated as "asbestos bodies" were identified by light microscopy in almost all organs [19]. Asbestos bodies are iron coated fibers that constitute only a portion (10–30%) of the total fibers visible by light microscopy and about 1% of the fibers visible by electron microscopy [20].

Ambient air contains asbestos fibers and, in fact, the general population carries a "background level of asbestos in their lungs" whose burden may attain about 10^6 ^fibers/g of dry lung tissue [20]; for occupationally exposed people, this burden may be about two orders of magnitude higher [20]. Asbestos fibers can potentially be dragged by bulk flows of water within and among compartments that represent porous systems where water circulates down pressure gradients. Before dealing in detail with the potential routes for translocation of asbestos fibers from the lung to other body compartments, it might be useful to consider the physical features of the motion of particulate dragged in biological fluids.

**Figure 2:**
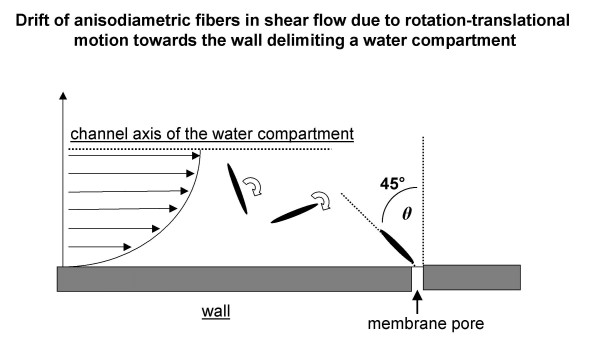
Continuous rotation, tumbling and oscillation result in drift of anisodiametric particles towards the wall delimiting the compartment (e.g. endothelial membrane). At the shear rate close to the wall, the lowest rotational velocity is attained at an angle *θ *of 45° relative to the wall; thus, a particle being driven by convective flow has a greater probability to hit the wall at this angle.

The flow of water in blood and lymphatic capillaries, across biological barriers (endothelium, mesothelium) and within the porous extracellular matrix can be considered of Poiseuille type with extremely low Reynold's number (< 0.005) [21]. As depicted schematically in Fig. 2, the motion of anisodiametric particles having density greater than that of the surrounding fluid (the average density of asbestos fibers is of the order of 2.5 g/cm^3^) is characterized by continuous rotation, tumbling, oscillation and drift even at very low Reynold's number. Furthermore, particle motion is altered by a hydrodynamic interaction in the proximity of the surface delimiting the channels of the porous medium. A typical phenomenon resulting from such a complicated motion pattern, is the translational drift of the particles towards the walls delimiting the channels (Fig. 2); this drift occurs at a faster rate with increasing anisodiametry of the particles [21]. Furthermore, the rotational velocity of an anisodiametric particle near the walls is not constant. Indeed, it varies as a function of the angle formed by the particle major axis with the wall. At the shear rate close to the wall, the lowest rotational velocity is attained at an angle of 45° relative to the wall; thus, a particle being driven by convective flow has a greater probability to hit the wall at this angle. Since the fluid streamline vector acting on the particle extremity farther from the wall is greater, the rotational vector might aid in the impact of the particle against the wall, similarly to a flying javelin falling on the ground. Therefore, drift eventually causes particles to hit the wall at an angle that might favour the translocation across the wall itself; as an example, the case of the endothelial membrane, either through a membrane pore or by causing a membrane discontinuity. Although not specifically modelled on physical grounds, one may presume that drift and translocation are different for chrysotile fibers, that are curly and flexible, in contrast to the amphiboles that are rod-like rigid fibers [22].

### Fluid dynamic pressure gradients in physiological condition and in interstitial lung edema

In physiological conditions, pulmonary interstitial pressure ranges ~ -10 cmH_2_O [6] and a net pressure gradient exists to cause filtration from microvessels to the lung interstitium. A small pressure gradient is also present to drive water from the pleural space to the lung interstitium across the visceral pleura [5]. Both trans-endothelial and trans-pleural flows are low due to the physiologically low permeability of these membranes [23,24].

When interstitial lung edema develops, common fluid dynamic features occur regardless of the model of edema studied, namely: an increased plasma volume (so called hydraulic non inflammatory model), i.v. elastase injection (inflammatory model), for hypoxia exposure (a mixture of hydraulic and inflammatory model) [25]. Following increased microvascular filtration, interstitial pressure increases from -10 up to about 5 cm H_2_O for a minor increase in extravascular water not exceeding 10%. Thus, interstitial lung edema represents a largely sub-clinical condition, due to the fairly low compliance of the interstitial matrix. The increased parenchymal stresses, that act as a "tissue safety factor" against further filtration, also entailed: 1) fragmentation of chondroitin and heparansulphate proteoglycans (belonging to matrix and basement membrane, respectively) due to loosening of non-covalent bonds and activation of tissue MMPs [25]; 2) increased expression of pro-inflammatory cytokines [26], 3) signalling-transduction in lung cells (mostly endothelial) in the process of matrix remodelling and deposition [27-30]. Another important consequence of the increase in interstitial pressure is the reversal of the trans-endothelial and trans-mesothelial pressure gradients allowing reabsorption of edema fluid.

### The impact of asbestos exposure on lung fluid dynamics

Experimental investigations and morphological studies on workers' lungs exposed to asbestos have shown early alveolar lesions reported as "asbestos alveolitis" [12,31] with local edema, microhemorrages and transformation of macrophages into "siderocytes". The inflammatory reaction may differ among lung regions reflecting the differences in fibers accumulation. In fact, fiber accumulation is greater in the cranial regions of the lung because the airway path to reach the alveolar compartment is shorter [32]. In these regions, morphometric analysis revealed a modest thickening of the alveolar septa due to extracellular matrix deposition and to a mild degree of interstitial edema [32].

Asbestos exposure triggers production of reactive oxygen species that leads to lipid peroxidation. This, in turn, affects the expression of genes intrinsic to inflammation [33]. Asbestos exposure also leads to depletion of extracellular superoxide dismutase thus increasing the susceptibility to reactive oxygen species [34]. Furthermore, a condition of inflammation was demonstrated by morphometric analysis of rat lungs exposed to an asbestos dose comparable to that in the work environment [35]. Asbestos exposure also caused an increased activity of metalloprotease MMP-9 temporally related to lung inflammatory reaction developing since day 1 after exposure. Conversely, the activity of metalloprotease MMP-2 increased at later time points (since day 7) corresponding to development of fibrosis [36]. It appears conceivable that following activation of MMPs, the loss of integrity of the extracellular matrix would confer greater mobility to asbestos fibers thus enhancing their translocation. The myeloperoxidase status was also shown to modulate the inflammatory reaction following asbestos exposure [37]. Asbestos exposure elicits stimulation of multiple cell-signaling pathways involving "a myriad of transcription factors and their cross-talk" [22], including the airway epithelial NF-kB that plays an important role in inflammatory response and cell proliferation [38].

In summary, the bulk of evidence indicates that asbestos exposure elicits an inflammatory reaction in the lung. Although it appears conceivable that regional differences are encountered, the highest inflammation possibly occurs at the site of fiber deposition.

### Fiber translocation via pulmonary lymph flow

**Figure 3:**
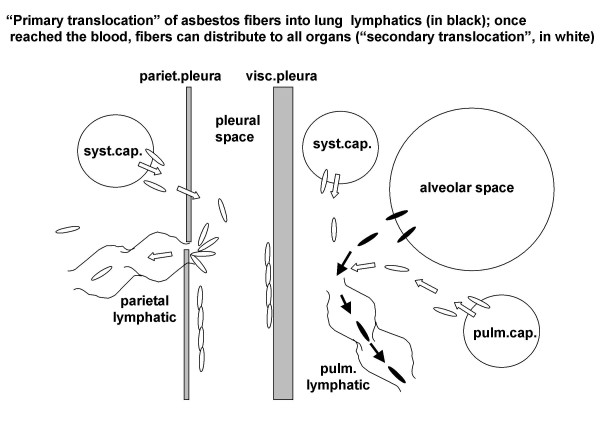
Asbestos fibers can be drained by convective flow into initial pulmonary lymphatics ("primary translocation", black particles). Once reached the blood through the lymphatic system, asbestos fibers can potentially translocate to all organs ("secondary translocation", shown by white particles) dragged by water fluxes down pressure gradients (white arrows).

Initial pulmonary lymphatics act as a reciprocating pump, i.e. generating intraluminal pressure oscillations to cause unidirectional suction-propulsion of the liquid thanks to unidirectional valves [4]. We shall call "primary translocation" (shown in Fig. 3 by black particles and black arrows) the movement of asbestos fibers into the lung and their clearance by convective flow into initial pulmonary lymphatics. The clearance of asbestos fibers from lung interstitium through the lymphatic system is a relatively slow phenomenon and in fact particles are eventually trapped in the tracheo-bronchial lymph nodes that become "reservoirs of retained material" [39]. Interestingly, the concentration of asbestos fibers in the lymph nodes is 2–3 fold higher than in the lung [40]. This finding can also be explained on the basis of water reabsorption occurring in the lymph nodes; indeed, the postnodal lymph flow is about 50% of the prenodal one and, correspondingly, protein concentration is about two fold higher. Lymphatic flow increases when interstitial pressure increases as a consequence of inflammation. Once reached the blood through the lymphatic system, asbestos fibers can potentially reach all organs via "secondary translocation", shown by white particles in Fig. 3: fibers are dragged by water fluxes down pressure gradients (white arrows in Fig. 3), to the extent that they can permeate the membranes separating the compartments and circulate in the extracellular spaces.

### Trans-endothelial fibers translocation

Most of microvascular exchanges occur via the porosity of the paracellular route (pores of ~5 nm and density of ~30/μm^2 ^and large pores of ~50 nm with a much lower density, ~1 large pore for 5000 small pores). Membrane sieving ought to be expected for any asbestos fiber whose hydrodynamic radius exceeds the size of the largest pores. The approximate hydrodynamic radius of the smallest asbestos fibers can be estimated from Stokes-Einstein relations (assuming a prolate ellipsoid shape). An equivalent radius < 50 nm can be estimated for the smallest nanoparticles with a diameter of 20 nm and a major to minor axis ratio < 5. So, the smallest asbestos particles can potentially permeate the largest endothelial membrane pores that are of about 50 nm. However, inflammation is expected to decrease steric hindrance as it increases the permeability of the endothelial barrier due to proteolysis of the intercellular link proteins mediated by the activation of metalloproteases [36].

**Figure 4:**
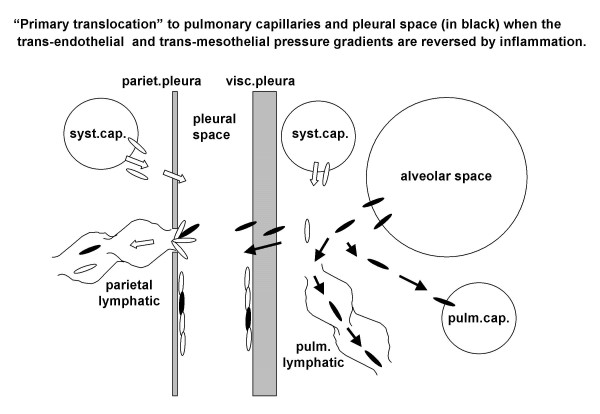
"Primary translocation" of asbestos fibers in pulmonary capillaries and across the visceral pleura occurs as long as the increase in pulmonary interstitial pressure due to inflammation is such as to reverse the pressure gradient across the pulmonary capillaries and the visceral pleura (black particles and black arrows).

For fibers residing in the lung interstitium a "primary translocation" to blood appears possible as long as the degree of interstitial edema, dependent upon the increase in microvascular permeability, leads to an increase in interstitial pressure high enough to generate an interstitium to capillary pressure gradient (black particles and black arrows in Fig. 4). Given the low compliance of the pulmonary interstitium, it is likely that even a modest increase in microvascular permeability leads to an increase in interstitial pressure that in turn causes a reversal of trans-endothelial pressure gradients for pulmonary capillaries. No reversal of trans-endothelial pressure can occur in systemic capillaries where pressure is higher than in pulmonary capillaries.

Over time, the inflammatory events evoked by asbestos exposure develop towards interstitial pulmonary fibrosis and it is conceivable that deposition of fibers would result in decreased permeability of microvessels and decreased porosity of the interstitial matrix. These events cause a hindrance to water flows and, correspondingly to flow driven fibers translocation. In this condition, the action of macrophages may become of relative greater importance in the translocation process of asbestos fibers.

### Trans-mesothelial fibers translocation

Primary translocation of asbestos fibers to the pleural cavity also requires a pressure gradient (black particles and black arrows in Fig. 4). As discussed above, this occurs if the degree of inflammation raises lung interstitial pressure up to ~5 cm H_2_O. Fiber translocation across the visceral pleura would be hindered by its thickness (~700 μm) and the prompt asbestos-induced fibrous deposition. The other route for pleural translocation of asbestos fibers is down the physiological pathway of pleural fluid formation at the parietal pleural level (shown in white in Fig. 3 and 4) [4]. The overall resistance encountered through this pathway is relatively high as it is represented by the sum of two resistances placed in series: the endothelium plus the mesothelium [5]. Yet, the parietal mesothelium has pores of relatively large equivalent radius, ~150–200 nm [5], compared to fiber hydrodynamic radius. The main pathway for pleural fluid drainage is through the lymphatic stomata of the parietal pleura and these are very abundant on the diaphragmatic surface and in the mediastinal region [41]. Accumulation of anisodiametric particles at stomatas entrance confirms a pleural flow driven translocation of asbestos fibers and also suggest a geometric hindrance for the fibers to permeate the initial lymphatic channels (as shown in Fig. 3). Parietal pleural plaques develop at the sites of stomatal opening. Accumulation of the smallest asbestos fibers also occurs at these sites (black spots) [42,43]. The hypothesis that pleural plaque pathogenesis is based on retrograde lymphatic drainage of asbestos fibers from the mediastinal lymph nodes to parietal pleura presently lacks a fluid dynamics based interpretation [44].

In the parietal pleura [40], only ultrathin, mostly ultrashort, fibers were observed [43,45]. The ultrathin fibers have clearly more chances to reach the pleural compartment due to a lower steric hindrance. In this respect it is of relevance to recall that using the ashing technique, high-resolution analytical electron microscopy allowed to establish that in malignant human mesothelioma tissues about 93% of the asbestos fibers, most of them of chrysotile type, were smaller than 0.25 μm in width [3]. The authors concluded that short, thin, asbestos fibers appear to contribute to the causation of human malignant mesothelioma. This recent view contrasts with the previous hypothesis, which was based on animal studies, that long and thin asbestos fibers (>or = 8 μm in length and < or = 0.25 μm in width) are strongly carcinogenic to induce pleural malignant mesothelioma, while shorter fibers would pose lesser risk [46].

The presence of asbestos fibers either free in pleural fluid or adsorbed on the mesothelial surface is likely to alter the delicate mechanism assuring pleural lubrication. Friction between pleurae is minimal under physiological conditions due to surfactant phospholipids secreted by mesothelial cells adsorbed on pleural surfaces [47]. Hydrophobic palmitoyl chains on surfactant molecules allow reciprocal sliding by maintaining a minimum film of fluid between the pleural surfaces [47] due to repulsive action of negative charges and keep the coefficient of friction at a surprisingly low value (~0.02). The microfluidics of pleural fluid is critically affected by the "porosity" and "tortuosity" of the pleural space [48] and the existence of fibers within the pleural space or adsorbed on pleural surfaces is likely to interfere with lubrication. Furthermore, mesothelial function is likely altered either directly or indirectly by the exposure of the lung to asbestos fibers. Indeed, in an animal model of asbestos exposure [49] the growth phase of mesothelial cells in the visceral pleura was a very early and marked phenomenon depending upon cytokine release by lung cells, essentially unrelated to pleural fibers deposition. The same study reported that the increase in pleural macrophages in response to asbestos inhalation was 5 fold greater than that of alveolar macrophages.

Exposure to asbestos inhalation was shown to trigger cell proliferation and fibrotic deposition in the visceral pleura [49]. Other observations in an experimental model [50] claim that a minimum amount of fibers could be found in pleural lavage fluid (crocidolite 0,5 μm diam, 10 μm length) after 1 week and this translocation was aided by an increase in permeability of the visceral pleura due to asbestos- induced NO formation.

Distribution of asbestos fibers was also demonstrated for the peritoneum, the pericardium and the tunica vaginalis of the testicle. Localization of fibers to the peritoneum may occur through filtration from blood capillaries of either the parietal or the visceral mesothelium. A potential route for asbestos fibers translocation between the serous cavities might be envisioned by considering that the muscular part of the diaphragm is extremely rich in lymphatic stomata both on pleural and peritoneal side, and these strain into a common submesothelial lacunar system [51,52] although valves should prevent such fluxes [53].

### Biopersistence of asbestos fibers in the lung

Studies on biopersistence in the lung tissue show that the half time for residence of amphiboles in the lung ranges from years to decades and they are chemically very stable, they do not fragment into shorter fibers although they can split longitudinally [20]. The estimated half time for chrysotile is much shorter (of the order of a few months) due to leaching, macrophage removal and fragmentation. In fact, progressive fragmentation represents the reasonable interpretation for the finding that the half time of chrysotile fibers is inversely proportional to fibers size (16 days for 20 μm fiber and 107 days for < 5 μm fiber) [54]. The long biopersistence in the lung can be attributed to fiber translocation by two routes, from the alveoli and from the blood; furthermore the development of fibrosis may result in a progressive greater hindrance to fiber translocation due to increased flow resistance of the porous interstitial matrix.

## Conclusion

**Figure 5:**
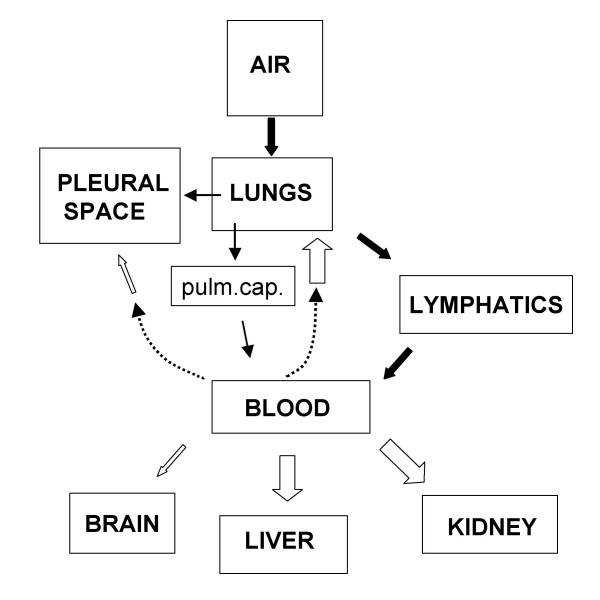
Block diagram to show primary (black arrows) and secondary (white arrows) asbestos fibers translocation. Size of arrows for secondary translocation are representative of accumulation. Accumulation increases in proportion to blood perfusion (lung, kidney and liver) and microvascular permeability (liver), while it is lower in brain and pleural space due to low permeability of the blood-brain barrier and of the endothelial-parietal mesothelium complex, respectively.

Asbestos fibers are found basically in all organs in subjects exposed to asbestos [55]. The block diagram in Fig. 5 shows primary (black arrows) and secondary (white arrows) asbestos fibers translocation within the body. The size of the arrows for secondary translocation are representative of asbestos fibers accumulation. The lowest concentration of asbestos fibers were found in the brain and pleural space [56] reflecting low permeability of the blood-brain barrier and of the endothelial-parietal mesothelial complex, respectively. Interestingly, in pleural mesothelioma, the most representative asbestos fibers are ultrathin ones [3], in line with a lower steric hindrance, compared to longer fibers, to be dragged down water fluxes. Fiber deposition was found relevant in the kidney and in the liver [56]. In the kidney this could be explained on the basis of a high blood perfusion and microvascular pressure. In the liver, this finding is in line with a high microvascular permeability of the liver sinusoids. The higher fiber concentration in the lung interstitium, compared to other organs, may result from two translocation routes as fibers can reach the lung interstitium both from the alveoli and from the blood.

In conclusion, the translocation of asbestos fibers within the body can be interpreted as a water flow driven process aided by the high biopersistency of asbestos fibers. Given the average size and aspect ratio of the asbestos particles, it is difficult to envision their translocation through the porous extracellular interstitial space and through the membrane barriers separating compartments. On the one hand, translocation may be aided by the increase in permeability of the membranes due to asbestos-induced inflammation and by the motion features of particulates in Poiseuille flow at low Reynolds numbers; on the other, translocation is likely hindered by fibrosis that actually increases interstitial flow resistance. The net result is a slow translocation process for asbestos particles developing over decades of life.

## Competing interests

The author(s) declare that they have no competing interests.

## Authors' contributions

GM and GC together conceived the review: GM on the basis of his scientific contribution on physiology and pathophysiology of fluid dynamics in the pleuro-pulmonary compartments; GC, as a professor of occupational health, having contributed in particular to the study of the correlation between pleural mesothelioma and asbestos exposure.

FM and GS contributed to the physical part dealing with motion of particles in Poiseuille flow regimen at low Reynolds number. All authors read and approved the final manuscript.
